# Massive thrombosis and phlegmasia cerulea dolens while taking rivaroxaban: case report and review

**DOI:** 10.1590/1677-5449.200036

**Published:** 2021-04-28

**Authors:** Diego Chemello, Larissa Rosa, Amanda Faria de Araujo, Pedro Cargnelutti de Araujo, Luiz Carlos Carneiro Pereira, Suélen Feijó Hillesheim, Marco Aurélio Lumertz Saffi

**Affiliations:** 1 Universidade Federal de Santa Maria – UFSM, Centro de Ciências da Saúde, Departamento de Clínica Médica, Santa Maria, RS, Brasil.; 2 Universidade Federal de Santa Maria – UFSM, Programa de Pós-graduação em Gerontologia, Santa Maria, RS, Brasil.; 3 Universidade Federal de Santa Maria – UFSM, Santa Maria, RS, Brasil.; 4 Hospital de Clínicas de Porto Alegre – HCPA, Porto Alegre, RS, Brasil.

**Keywords:** anticoagulants, venous thrombosis, sinus thrombosis, intracranial, anticoagulantes, trombose venosa, trombose dos seios intracranianos

## Abstract

Our study describes a fatal case of phlegmasia cerulea dolens and massive venous thrombosis in a patient taking rivaroxaban regularly to treat cerebral venous sinus thrombosis. Blood tests samples were positive for lupus anticoagulant. The unique evolution of the case, as well as the positivity for lupus anticoagulant, raises the possibility of an acquired hypercoagulation syndrome. We highlight the fact that the test recommended as the first line for lupus anticoagulant diagnosis (dilute Russell viper venom time) is the most affected by rivaroxaban, leading to a high prevalence of false-positive results. We also discuss potential diagnoses for the current case and review the current state-of-the-art of use of the novel oral anticoagulation agents in this unusual situation. So far, there are no recommendations to use such agents as first options in cerebral venous sinus thrombosis or in hypercoagulation syndromes.

## Resumo

Nosso estudo descreve um caso fatal de flegmasia cerúlea dolens e trombose venosa maciça em um paciente usando regularmente rivaroxabana para o tratamento de trombose de seio venoso cerebral. A investigação laboratorial foi positiva para o anticoagulante lúpico. A evolução única do caso aumenta a possibilidade de uma síndrome de hipercoagulabilidade adquirida, bem como a positividade para o anticoagulante lúpico. Destacamos o fato de que o teste recomendado como primeira linha para o diagnóstico de anticoagulante lúpico (veneno de víbora de Russel diluído) é o mais afetado pela rivaroxabana, levando a uma alta prevalência de resultados falso-positivos. Também discutimos os potenciais diagnósticos para o presente caso e revisamos o estado da arte atual dos novos agentes de anticoagulação oral usados nessa situação incomum. Até o presente momento, não há recomendações para o uso de tais agentes como primeira opção na trombose de seios venosos cerebrais ou nas síndromes de hipercoagulação.

## INTRODUCTION

Cerebral venous sinus thrombosis (CVST) is a rare disorder with an annual estimated incidence of up to 4 cases per million and accounting for approximately 0.5% of all patients presenting with stroke-like symptoms.^1^ The current treatment for CVST is heparin, followed by vitamin K antagonists (VKA) for 3-12 months.^2^ Direct oral anticoagulants (DOACs), or non-vitamin K oral anticoagulants (DOACs), have emerged as an alternative to VKA, in particular due to the lower risk of intracranial and fatal bleeding. Small case series and case reports have described successful CVST treatment with DOACs. More recently, the exploratory clinical trial RE-SPECT CVT determined the short-term safety and efficacy of dabigatran for preventing recurrent venous thromboembolisms (VTEs) in patients with CVST. We report a unique and fatal case of recurrent massive thrombosis with development of phlegmasia cerulean dolens within 30 days of CVST treatment, while the patient was taking rivaroxaban regularly. The Research Ethics Committee approved this study (protocol N^o^. 23081.004589/2018-03).

## CASE DESCRIPTION

**Figure 1:**
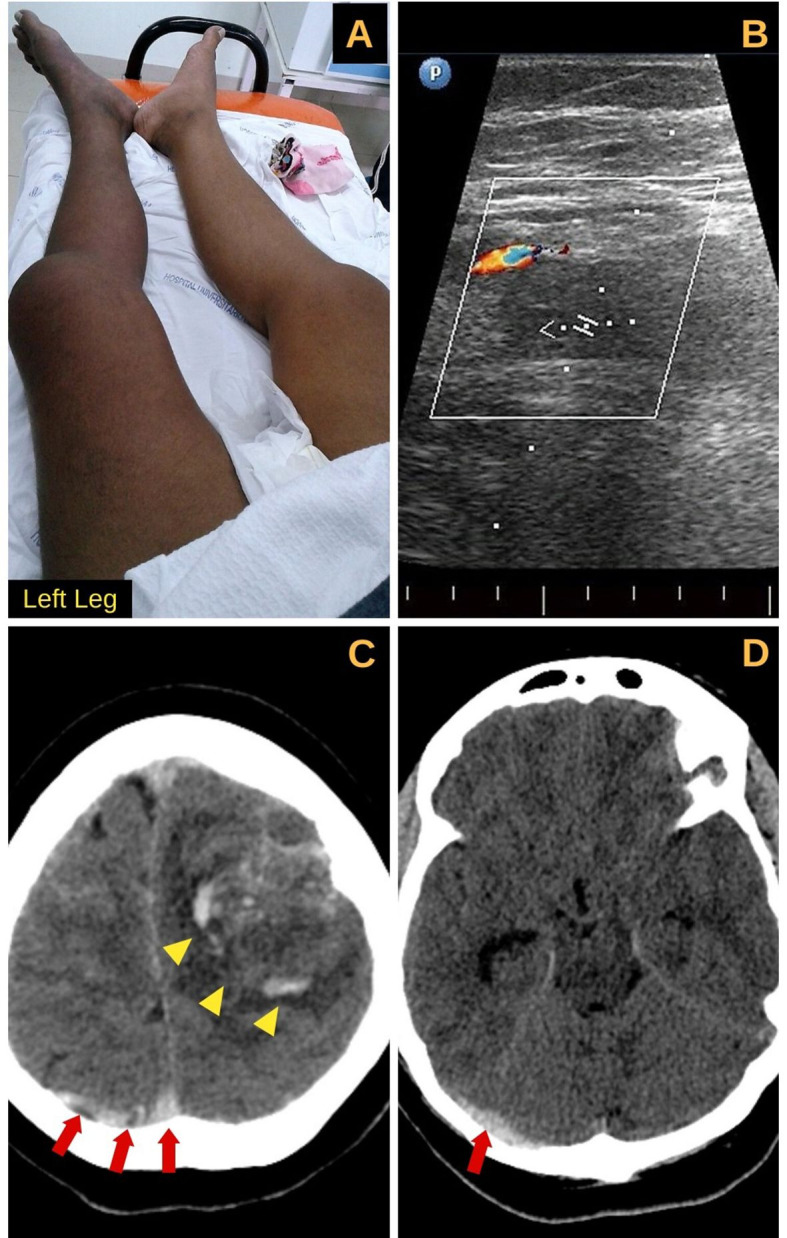
Images from Case: Massive left leg edema with cyanosis (A). Venous ultrasound with Doppler of the common left iliac vein showing no venous flow and absence of compression; signs compatible with deep vein thrombosis (B). Non-contrast enhanced computed tomography scan showing signs of cerebral venous thrombosis. The images show dense clot signs in the sagittal and right transverse sinuses (red arrows) (C, D) and left temporoparietal hemorrhage with surrounding edema (yellow arrowheads) (C).

A 44-year-old, hypertensive, African American female presented to the emergency department (ED) with recent onset of pain and bilateral leg edema in conjunction with acute change in mental status. Physical examination showed somnolence, hypotension (blood pressure 80/40 mmHg), and tachycardia (136 bpm). There was leg swelling and cyanosis, more prominent on the left side (Figure 1A). After an unsuccessful volume resuscitation attempt, she was started on norepinephrine with progressively higher doses and only partial hemodynamic improvement. She rapidly progressed to respiratory failure, requiring mechanical ventilation and intensive care unit (ICU) support.

Her recent medical history was notable for a 60-day diagnosis of CVST, compromising the dural venous and the sigmoid sinuses (Figure 11D). The thrombosis was initially associated with left frontotemporal bleeding. Neurological assessment indicated anticoagulation with unfractionated heparin (UFH). She was then maintained on rivaroxaban (20mg/day), because of difficulties with warfarin control. She had been followed by the Home Care Service and had good compliance with medical treatment.

At the ED, laboratory exams revealed metabolic acidosis. There was leukocytosis with left shift (28.830 cells/mm^3^, 13% immature), and renal impairment (creatinine: 2.09 mg/dl). Coagulation tests showed normal platelet counts (278,000 cells), elevated prothrombin time (35.9%; international normalized ratio [INR]: 1.91), and normal activated partial thromboplastin time (27.4 sec). Lactate levels were elevated (4.8 mmol/l).

Emergency ultrasound confirmed severe bilateral deep vein thrombosis with right common femoral and left proximal iliac involvement (Figure 1B). A diagnosis of phlegmasia cerulean dolens (PCD) was made. Despite the previous and regular rivaroxaban use (which was also suggested by the elevated prothrombin time), systemic anticoagulation with UFH was started. Laboratory tests for hypercoagulable states were collected, revealing positive results for lupus anticoagulant (LA), according to the dilute Russell viper venom time (DRVVT).

The patient’s hemodynamic instability precluded vascular CT for confirmatory diagnosis of CVST recurrence and assessment of the extent of deep venous thrombosis. At this point, a vascular surgical intervention was planned for extensive thrombosis. Despite escalating doses of vasopressors and systemic anticoagulation, the patient developed refractory shock and died a few hours later with no opportunity for intervention.

## DISCUSSION

We present a unique case report of a patient who developed PCD with a rapid fatal outcome while regularly taking rivaroxaban. Direct oral anticoagulants are approved for stroke prevention in patients with non-valvular atrial fibrillation (AF) and for VTE treatment.^3^^,^ 4 These medications offer many advantages over VKA, such as predictable dose-response, fewer drug and food interactions, and no need for laboratory monitoring of the INR or other coagulation tests. However, there is no current recommendation to choose DOACs as first-line treatment agents for CVST or for acquired hypercoagulable states, such as antiphospholipid syndrome (APS).

Long-term anticoagulation with VKA is the standard approach to prevention of CVST recurrence. Recurrence of thrombosis is also high (approximately 6.5% per year). Although thousands of patients have used and are using DOACs in everyday practice; little is known about the efficacy and safety of these agents in patients diagnosed with CVST. Current American Heart Association/American Stroke Association guidelines for the treatment of cerebral vein thrombosis dating back to 2011 endorse the utility of anticoagulation for treatment of cerebral vein thrombosis, but they do not support use of direct oral anticoagulants. Updated guidelines from the European Stroke Organization, endorsed by the European Academy of Neurology in 2017, also reject utilization of direct oral anticoagulants due to a lack of evidence.^2^^,^ 5

Geisbüsch et al.^6^ reported their experience with 7 CVST patients who were treated with the factor Xa inhibitor rivaroxaban, in comparison with 9 patients treated with phenprocoumon. These medications had similar clinical benefits.

Few case series have described successful results of CVST patients treated with dabigatran and apixaban.^7^^,^ 8 So far, the quality of evidence has been judged as very low because all studies were observational with a high risk of bias.^2^ Recently, results from the exploratory RE-SPECT CVT study endorsed the positive results of DOAC against VTE recurrence after a CVST episode. This is an international randomized, open-label multicenter trial that investigated the efficacy and safety of dabigatran etexilate versus dose-adjusted warfarin for a period of 24 weeks on a net clinical benefit endpoint of major bleeding and thrombotic events. The results showed no thrombotic events or deaths among patients on dabigatran.^9^ Dabigatran is a direct thrombin antagonist that has been proven to be efficacious and to have a good safety and tolerability profile when used for stroke prevention in patients with atrial fibrillation^10^ as well as when used for treatment and prevention of recurrent deep venous thrombosis and pulmonary embolism.^11^ Despite the promising results with the direct thrombin inhibitor dabigatran in CVST treatment, definitive conclusions are pending regarding the superiority of this agent.

The unusual presentation and severity of thrombosis in the present case, as well as the single positive test for LA antibodies, highlight the possibility of APS. According to international consensus statements, APS is characterized by venous or arterial thrombosis and/or pregnancy morbidity with persistent positivity (two positive serum or plasma tests at least 12 weeks apart) of one of three serologies: (1) LA, (2) anti-cardiolipin antibodies, or (3) anti-β2 glycoprotein 1 antibodies.^12^ Despite the positive LA test, it is impossible to establish a definitive diagnosis in the present case, since the laboratory test criterion was not met. Moreover, there are several reports of false-positive and unreliable LA results in patients taking rivaroxaban. Martinuzzo et al.^13^ confirmed that the test recommended as the first line for LA diagnosis (DRVVT) is the most affected by rivaroxaban, leading to a high prevalence of false-positive results.

Considering the uncertainties in the present case, the initial decision to switch from warfarin to DOAC looks controversial at best. Although preliminary reports have supported use of DOACs in APS patients with a history of venous thrombosis and a target INR of 2-3, cases of recurrent thrombosis while on these agents have been described.^14^^-^ 16 Dufrost et al.^17^ recently conducted a systematic review regarding APS patients treated with DOACs, most treated with rivaroxaban. They concluded that these medications are not effective in all APS patients and should not be used routinely. TRAPS was a randomized, open-label, noninferiority study investigating rivaroxaban compared to warfarin in high-risk patients (with triple-positive antiphospholipid antibody tests) with thrombotic APS.^18^ The trial was terminated prematurely after enrollment of 120 patients (59 randomized to rivaroxaban and 61 to warfarin) because of an excess of events among patients in the rivaroxaban arm. While there were 7 (12%) thromboembolic events in patients randomized to rivaroxaban, no events were recorded in those randomized to warfarin.^19^

ASTRO-APS is a prospective phase IV randomized open-label blinded event pilot study that will randomize patients with a clinical diagnosis of APS receiving therapeutic anticoagulation to either adjusted-dose warfarin or apixaban.^20^ The study initially planned to use 2.5mg twice a day, but a prespecified Data Safety Monitoring Board (DSMB) review was convened after enrollment of 25 patients and recommended the protocol be modified to use apixaban 5 mg twice a day. After an additional 5 patients had been enrolled, a possibly higher than expected rate of stroke among patients with a history of stroke randomized to apixaban was observed. This led to a decision to only enroll patients with clinical APS and a history of venous thrombosis (excluding patients with prior arterial thrombosis from enrollment) and to obtain a brain magnetic resonance imaging for all otherwise eligible candidates, then enroll those patients without radiographic evidence of prior stroke or white matter changes disproportionate for patient age. The ASTRO-APS is actively screening and endeavors to enroll 200 patients.^21^

RAPS was a randomized, open-label non-inferiority trial investigating rivaroxaban versus warfarin use in thrombotic APS (with or without lupus erythematosus). The primary outcome was percentage change in endogenous thrombin potential (ETP) from randomization to day 42, with non-inferiority set at less than 20% difference from warfarin in mean percentage. Although ETP for rivaroxaban did not reach the non-inferiority threshold, there was no increase in thrombotic risk compared with standard-intensity warfarin.^22^

Additionally, some authors advocate that when deciding to use a DOAC in these patients, it might be preferable to choose one that has a twice-daily dosage (like apixaban or dabigatran). Despite little clinical evidence, twice-daily dosed DOACs (rather than once-daily dosed DOACs) may lead to more steady drug levels throughout the 24-hour period. This could in theory provide a more effective anticoagulant effect in these very prothrombotic patients.^23^

Finally, it is important to highlight that this case report presents an unfavorable patient outcome. More studies in this area are needed to identify more robust and effective approaches, although these are difficult to determine because of the uncommon occurrence of this type of clinical situation.
